# Integrated bioinformatics analysis of the shared molecular mechanisms between Parkinson’s disease and COVID-19

**DOI:** 10.1128/msphere.00908-25

**Published:** 2026-04-03

**Authors:** Yang Su, Hui Ma, Jiayuan Niu, Dongnan Hou, Liya Li

**Affiliations:** 1 Department of Anesthesiology, The Second Affiliated Hospital of Dalian Medical University 36674, Dalian, China; Instituto de Biotecnologia/UNAM, Cuernavaca, Morelos, Mexico

**Keywords:** Parkinson’s disease, COVID-19, CHI3L1, scRNA-seq, cell-to-cell communication

## Abstract

To investigate the shared molecular mechanisms between Parkinson’s disease (PD) and COVID-19 through integrated bioinformatics analysis and single-cell RNA sequencing (scRNA-seq). We conducted a comprehensive analysis of bulk RNA-seq data from publicly available databases, along with scRNA-seq data from brain tissues of COVID-19 patients. Differential expression analysis identified 725 differentially expressed genes (DEGs) in COVID-19 and 633 in PD samples. A total of 77 overlapping DEGs were identified, highlighting common pathways associated with neuroinflammation and dopaminergic neuron dysfunction. Gene Ontology and Kyoto Encyclopedia of Genes and Genomes analyses revealed significant enrichment in inflammation-related pathways. The protein-protein interaction network analysis identified CHI3L1 as a key gene linking PD and COVID-19. ScRNA-seq analysis revealed a significant increase in CHI3L1-expressing astrocytes in COVID-19 samples, indicating a potential mechanism by which COVID-19 may exacerbate PD symptoms. Furthermore, cell-cell communication analysis revealed enhanced interactions between astrocytes and microglia, excitatory neurons, or oligodendrocytes through signaling molecules such as phosphoprotein 1, CADM1, NCAM1, NRG, and NRXN1, suggesting that astrocytes play a central role in regulating neuronal excitability, synaptic plasticity, and immune responses in the context of COVID-19. These findings suggest a complex interplay between COVID-19 and PD, emphasizing the need for further investigation into the shared pathogenic mechanisms and potential therapeutic targets.

**IMPORTANCE:**

This study demonstrates the critical role of neuroinflammation and dopaminergic neuron damage in the shared pathogenesis of COVID-19 and Parkinson’s disease. CHI3L1 emerges as a key target, highlighting its potential involvement in modulating neuroinflammatory pathways and synaptic plasticity. The functional significance of CHI3L1, along with its pathological relevance, warrants further investigation through larger studies. Additionally, the active intercellular communication among astrocytes, microglia, and excitatory neurons underscores the profound impact of COVID-19 on neural circuitry. Collectively, these results provide important insights into the mechanisms driving the neurodegenerative consequences of COVID-19, emphasizing the need for continued exploration of therapeutic interventions and the long-term neurological effects of viral infection.

## INTRODUCTION

The global health impact of COVID-19 is profound, characterized by severe respiratory distress and the potential for multi-organ failure (1). Although new variants and waning immunity from vaccines or prior infections may lead to fewer severe cases, long-term complications are increasingly prevalent. Evidence shows post-COVID-19 conditions such as pulmonary fibrosis, myocardial injury, thrombotic events, and neurological impairments, including cognitive deficits and peripheral neuropathy (2). Notably, neurological complications have emerged as particularly prominent in the aftermath of COVID-19. Reports indicate a significant increase in the prevalence of ischemic stroke, brain hemorrhage, and seizures during the pandemic (3). Additionally, dementia, when associated with COVID-19, ranks as the second leading cause of COVID-related mortality (4). The overall prevalence of post-COVID-19 physical, cognitive, and emotional impairments is estimated to range between 9% and 63%, significantly higher than that observed in similar viral infections (5).

Viral infections have long been implicated as potential etiological or triggering factors in the pathogenesis of Parkinson’s disease (PD) (6). Previous studies have demonstrated that certain coronaviruses exhibit a notable affinity for the basal ganglia, which may contribute to the onset of PD (7). As a novel coronavirus, SARS-CoV-2 has also been suggested to play a role in PD occurrence and development. There was a case report of acute PD emerging after COVID-19 infection (8). Moreover, olfactory dysfunction, an early non-motor symptom of PD, is frequently observed in COVID-19 patients, suggesting a potential link between COVID-19 and an increased risk of PD onset (9, 10). Clinical investigations have further revealed that COVID-19 significantly exacerbates both motor and non-motor symptoms in individuals with PD, particularly in older adults and those with multiple comorbidities (10, 11). While COVID-19 may influence PD pathogenesis through several potential mechanisms, including damage to dopaminergic neurons, induction of neuroinflammation, and oxidative stress, the exact mechanisms and key therapeutic targets remain elusive (12–15). Further research is needed to elucidate the relationship between the two diseases.

Bioinformatics has emerged as a crucial tool for elucidating the complex relationships between diseases by analyzing large-scale omics data to uncover underlying mechanisms. This study aims to integrate and analyze gene data related to the pathogenesis of COVID-19 and PD sourced from public databases, thereby providing new insights into the shared biological mechanisms of these two conditions. Furthermore, we incorporated single-cell sequencing and intercellular communication analyses of brain tissues from COVID-19 patients to enhance our understanding of the connections between viral infection and PD. Ultimately, this research seeks to identify potential therapeutic targets and facilitate the development of dual prevention strategies.

## MATERIALS AND METHODS

### Bulk-seq data collection and analysis

The bulk-seq expression profile and relevant clinical data of PD and COVID-19 samples at various stages were collected from the Gene Expression Omnibus (GEO; https://www.ncbi.nlm.nih.gov/geo/), accession numbers GSE184950 and GSE182299.

### Analysis of DEGs by bulk-seq

The bulk RNA-seq expression profile was preprocessed through background correction, gene symbol transformation, and normalization using RStudio (ver. 4.2.0, https://www.r-project.org/). Limma package (16) was initially applied to distinguish differentially expressed genes (DEGs) between PD and COVID-19 samples. Significant DEGs were defined as those exhibiting an absolute log2 fold change (log_2_FC) greater than 1 and an adjusted *P* value < 0.05.

### Functional annotation analyses, PPI network construction, and module analysis

Subsequently, Gene Ontology (GO) and Kyoto Encyclopedia of Genes and Genomes (KEGG) pathway enrichment analyses were employed on DEGs by cluster Profiler (version 4.0.5) (17). Min overlap = 3 and Min enrichment = 1.5 were the screening conditions. The *P* value < 0.01 was considered significant. The protein–protein interaction (PPI) network was analyzed using the Search Tool for the Retrieval of Interacting Genes (STRING; http://string-db.org). An interaction with a combined score > 0.4 was selected, and the Cytoscape tools (https://cytoscape.org/) were used to estimate the hub genes with the molecular complex detection (MCODE) algorithm: K-core = 2, degree cutoff = 2, max depth = 100, and node score cutoff = 0.2.

### Single-cell transcriptome analysis

All analyses in the present study were performed using R software (version 4.1.1). The single-cell RNA sequencing (scRNA-seq) data were analyzed using the Seurat package (version 4.2). Low-quality cells with less than 300 or over 7,500 expressed genes, or over 25% unique molecular identifiers derived from the mitochondrial genome, were removed. We used canonical correlation analysis to correct for batch effects across data sets. The top 2,000 highly variable genes were identified using the Find Variable Features function in the Seurat package with default parameters.

### ScRNA-seq data collection, cell clustering analysis, visualization, and annotation

The Chromium 10× scRNA-seq expression profiles, along with clinical data from six subjects collected from healthy donors and COVID-19 patients at different stages, were retrieved from the GEO database. The gene expression matrix and associated clinical data were imported into RStudio (version 4.2.0) for further analysis using the Seurat package (18). Initial data preprocessing included filtering out low-quality single cells—defined as those expressing fewer than 300 genes, containing more than 10% mitochondrial transcripts, or more than 0.5% red blood cell transcripts. Additionally, genes expressed in fewer than three single cells were removed.

After filtering, the remaining data set was normalized. The top 5,000 highly variable features were selected using a variance-stabilizing transformation. These features were then subjected to *z*-score transformation, using a linear scaling method to center and standardize the data set. Principal component analysis (PCA) was subsequently performed to reduce the data’s dimensionality, focusing on the top 5,000 most variable genes. The first 20 principal components were utilized for further analyses based on the FindNeighbors algorithm with default parameters. A resolution parameter of 0.5, adjusted between 0.1 and 1 depending on the data set, was employed to identify cell clusters.

For non-linear dimensionality reduction and visualization of single-cell clusters, *t*-distributed stochastic neighbor embedding (t-SNE) was applied. Marker genes for each cluster were identified, and the top three significantly upregulated genes (adjusted *P* value < 0.05, minimum percentage > 0.25, and log_2_FC > 0.25) were displayed via heatmap. Cell type annotation for the identified clusters was performed using the SingleR algorithm in conjunction with the CellMarker database (http://bio-bigdata.hrbmu.edu.cn/CellMarker/index.html).

### Cell-cell communication analysis

Intercellular communication analysis was conducted using the CellChat package (version 1.5.0) for scRNA-seq data (19). Interactions between cells were deemed significant if the *P* value of ligand–receptor pair interactions was less than 0.05.

### Statistical analysis

Statistical analysis was performed using RStudio, with the significance testing methods dictated by the respective packages utilized. A two-sided *P* value of less than 0.05 was regarded as statistically significant.

## RESULTS

### Identification of DEGs in bulk RNA-seq data

**Fig 1:**
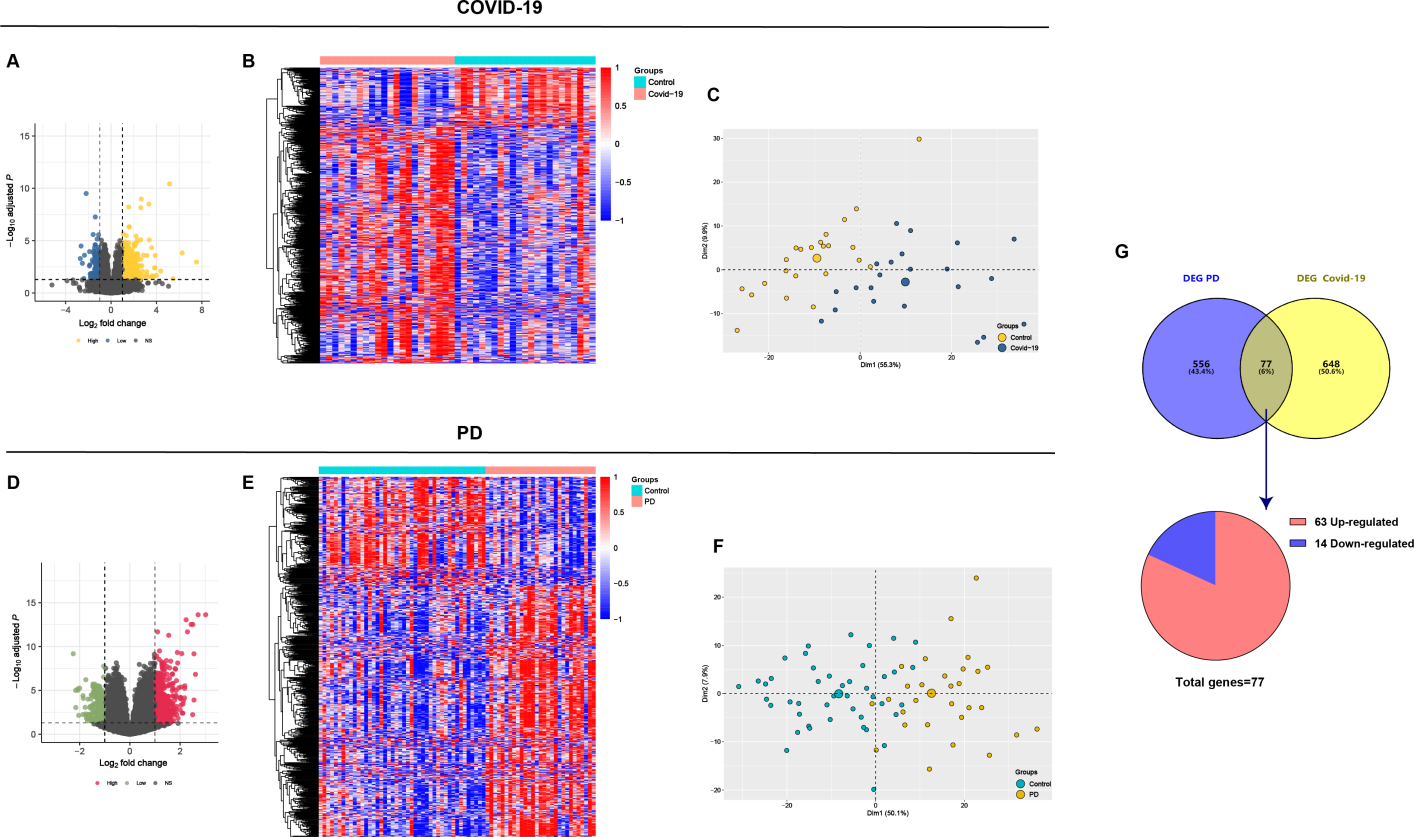
Analysis of common targets between COVID-19 and PD from two public databases. (**A and B**) Volcano plot and heatmap demonstrating an overview of the differential expression of all genes in panel B. (**C**) Principal component analysis (PCA) of all genes in COVID-19. (**D and E**) Volcano plot and heatmap demonstrating an overview of the differential expression of all genes in PD. (**F**) PCA of all genes in PD. (**G**) Venn diagram demonstrates the common DEGs of COVID-19 and PD. The threshold in the volcano plot was −log10 (adjusted *P*-value) > 2 and |log2 (fold change)| > 0.5. FDR was used (Benjamini Hochberg’s) for *P* value adjustment.

We conducted a comprehensive search of the NCBI GEO databases for bulk RNA-seq data derived from brain tissues of COVID-19 intensive care unit (ICU) specimens and PD specimens. Differential expression analysis identified 725 DEGs in COVID-19 (Fig. 1A and B) and 633 in PD specimens (Fig. 1D and E). PCA was applied to the normalized data (Fig. 1C and F), revealing 77 overlapping DEGs, including 63 upregulated and 14 downregulated genes (Fig. 1G). These DEGs were subsequently interrogated as potential biomarkers for the mechanistic linkage between the two conditions.

### Functional enrichment analysis of common DEGs in two diseases

**Fig 2:**
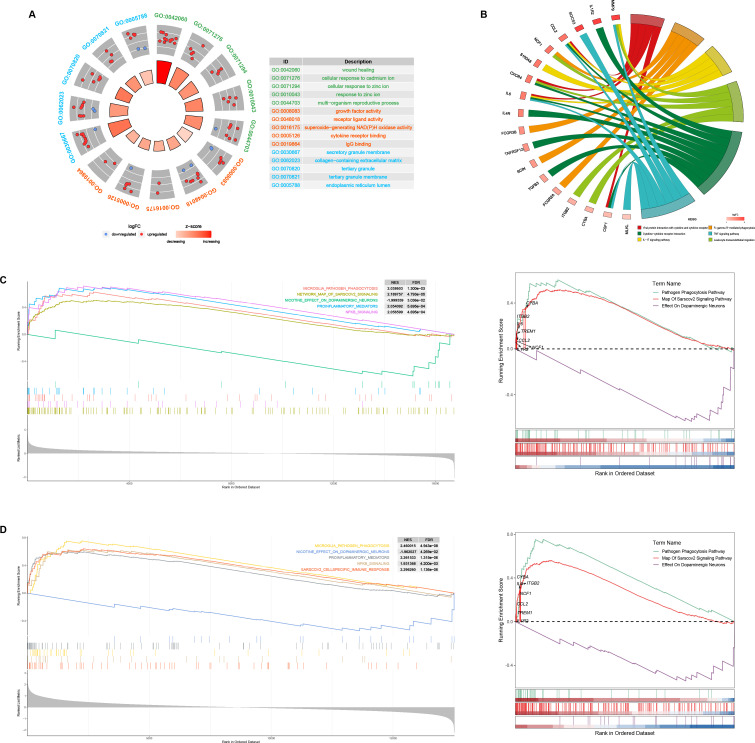
Comprehensive functional annotation of 77 risk genes. (**A**) GO functional enrichment analysis of the 77 genes (top five terms). (**B**) KEGG revealed the top six pathways enriched in the 77 genes. (**C**) GSEA annotation of the DEGs in COVID-19. (**D**) GSEA annotation of the DEGs in PD.

GO and KEGG enrichment analyses were conducted to elucidate the biological pathways associated with the shared DEGs. For the GO enrichment analysis, a total of 627 pathways reached statistical significance (*P* < 0.05). The top five pathways for each GO term—biological process (BP), cellular component, and molecular function—are presented in Fig. 2A. Notably, the common DEGs were primarily associated with biological processes such as wound healing, cellular responses to cadmium and zinc ions, growth factor activity, and receptor-ligand activity (Fig. 2A). KEGG pathway analysis revealed the top six significantly enriched pathways (Fig. 2B), including viral protein interaction with cytokine and cytokine receptors, Fc gamma R-mediated phagocytosis, cytokine-cytokine receptor interaction, tumor necrosis factor signaling pathway, IL-17 signaling pathway, and leukocyte transendothelial migration. Additionally, gene set enrichment analysis (GSEA) indicated a significant activation of proinflammatory mediators and suppression of dopaminergic neuron function in the brain tissues of both COVID-19 and PD specimens (Fig. 2C and D). Core DEGs concurrently mapping to COVID-19 and PD pathogenesis pathways (pathogen phagocytosis, SARS-CoV-2 signaling, and dopaminergic neuron) are also visualized in Fig. 2C and D. These findings align with the KEGG results, highlighting the role of inflammation-related pathways and secondary damage to dopaminergic neurons as potential links between COVID-19 and PD.

**Fig 3:**
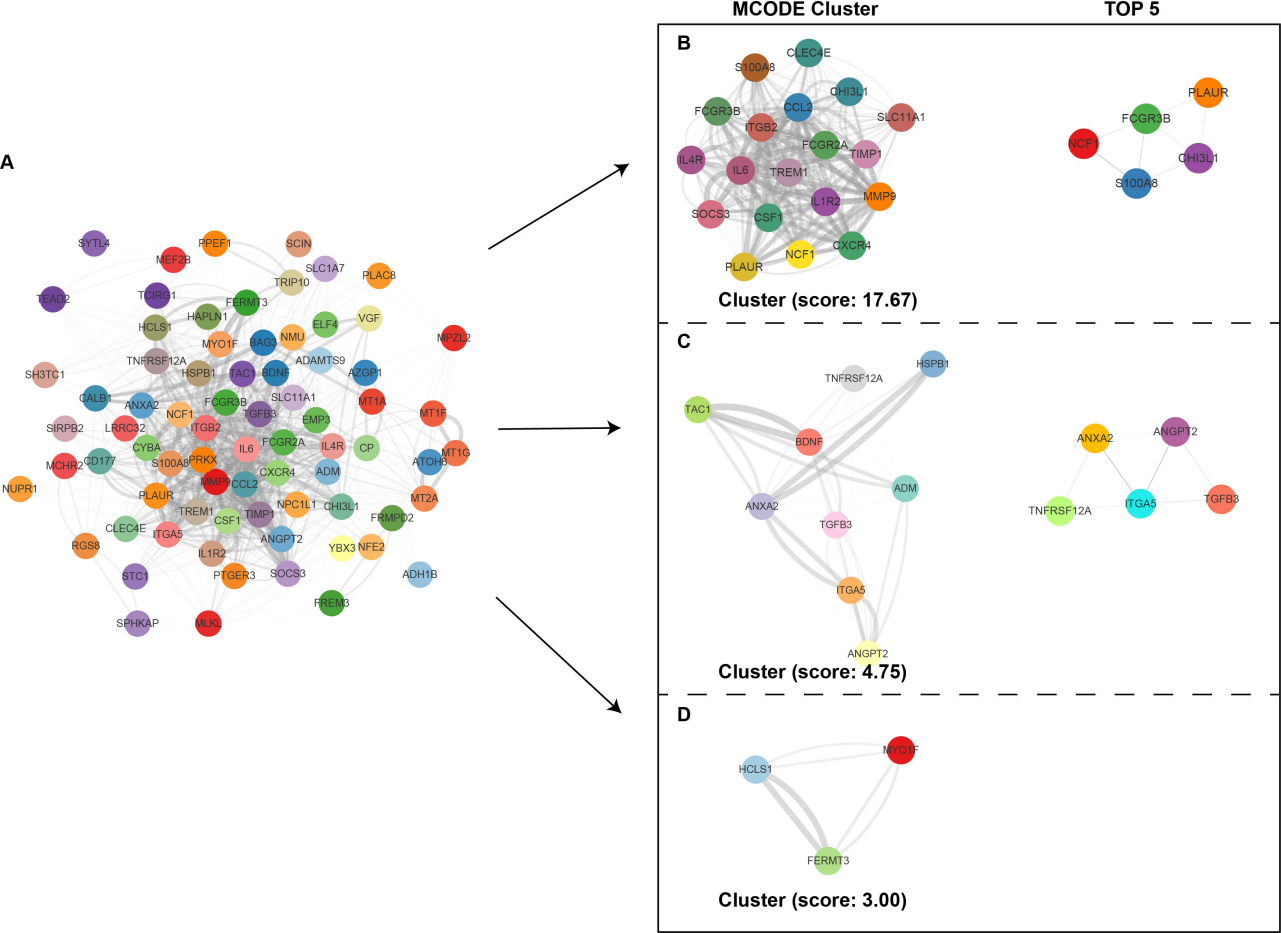
The PPI network and clusters analysis of common targets. (**A**) PPI network of 77 common targets. (**B–D**) Three significant gene clustering modules and enrichment analysis of the modular genes.

The PPI network of the shared DEGs was constructed using Cytoscape, with interactions having a combined score greater than 0.4. Stronger interactions are depicted by darker lines, indicating higher confidence (Fig. 3A). Using the MCODE plugin in Cytoscape, three densely connected gene modules were identified, and the top five genes from each module were selected as potential core genes linking COVID-19 and PD (Fig. 3B–D). Notably, 19 core genes were identified through the MCODE method, with *CHI3L1*, *S100A8*, *NCF1*, *FCGR3B*, and *PLAUR* occupying key positions within the core network, as determined by the maximal clique centrality algorithm (Fig. 3B).

### Identifying cell populations and molecular mechanisms in COVID-19 with scRNA-seq

**Fig 4:**
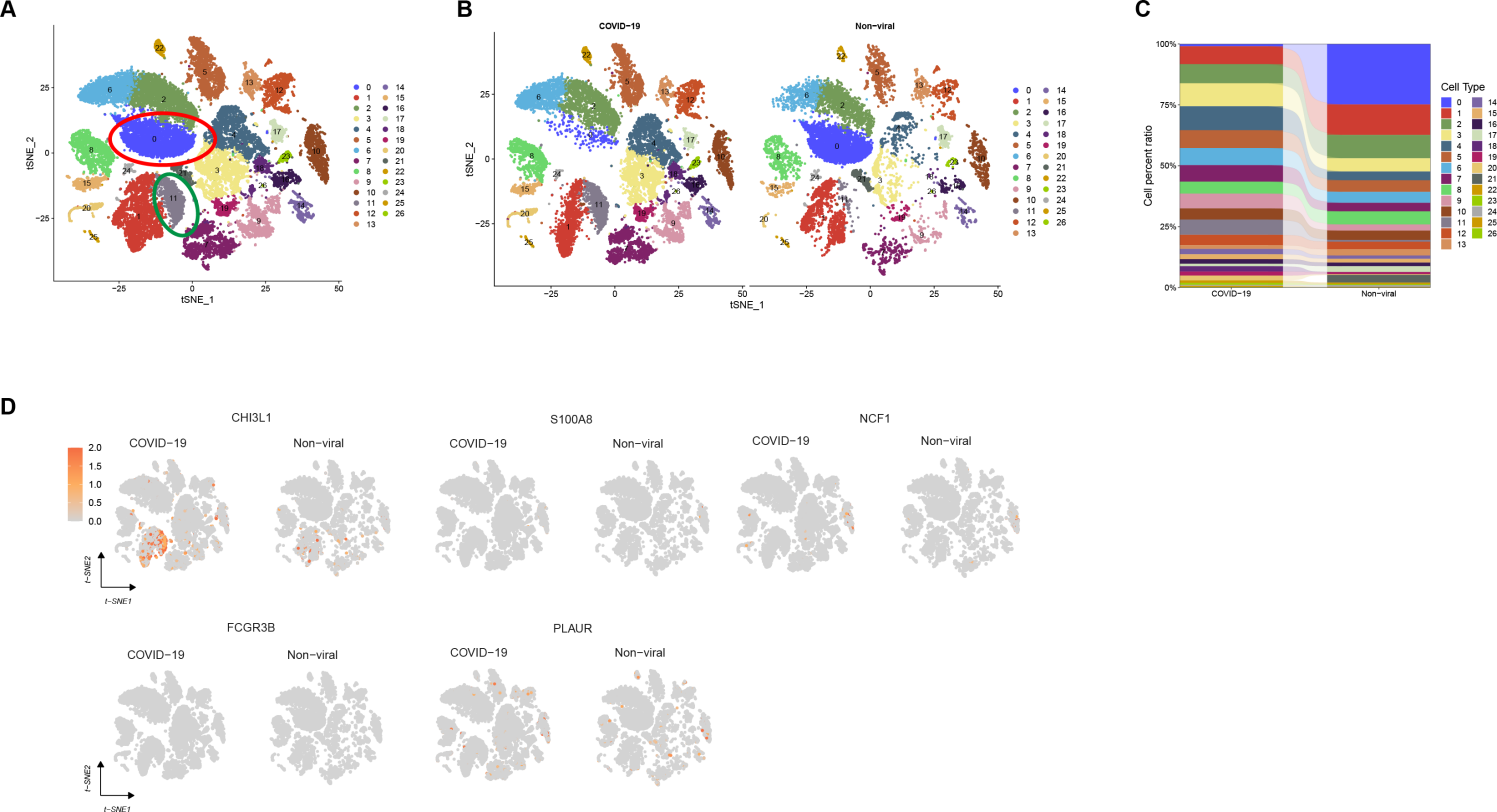
Cell typing and molecular mechanisms in COVID-19. (**A**) Identifying clusters of brain tissues from COVID-19 and non-viral samples. (**B**) t-SNE projection of brain tissues from the COVID-19 (left panel) and non-viral samples (right panel). Clusters are colored and labeled according to their inferred cell type identities. (**C**) The number of cells and box plots of the number of genes. (**D**) Expression levels for genes with cluster-specific patterns in COVID-19 and non-viral samples.

Database from GSE182298 was subjected to scRNA-seq from brain tissues of ICU specimens who died from severe COVID-19 and paired uninfected control specimens. The results showed that there was one kind of cell subpopulation in COVID-19 specimens. The analysis revealed a significant increase in the number of cells in subpopulation 11 and a significant decrease in the number of cells in subpopulation 0 in COVID-19 specimens (Fig. 4A through C). Among the core genes, only CHI3L1 was found to have higher expression in subpopulation 11 (Fig. 4D). Clinical studies had confirmed that CHI3L1 was involved in the pathogenesis of COVID-19 and was associated with adverse outcomes (20, 21). CHI3L1 is also an important biomarker for early detection of PD (22, 23), suggesting that CHI3L1 may be closely associated with an increased risk of PD following COVID-19 infection.

**Fig 5:**
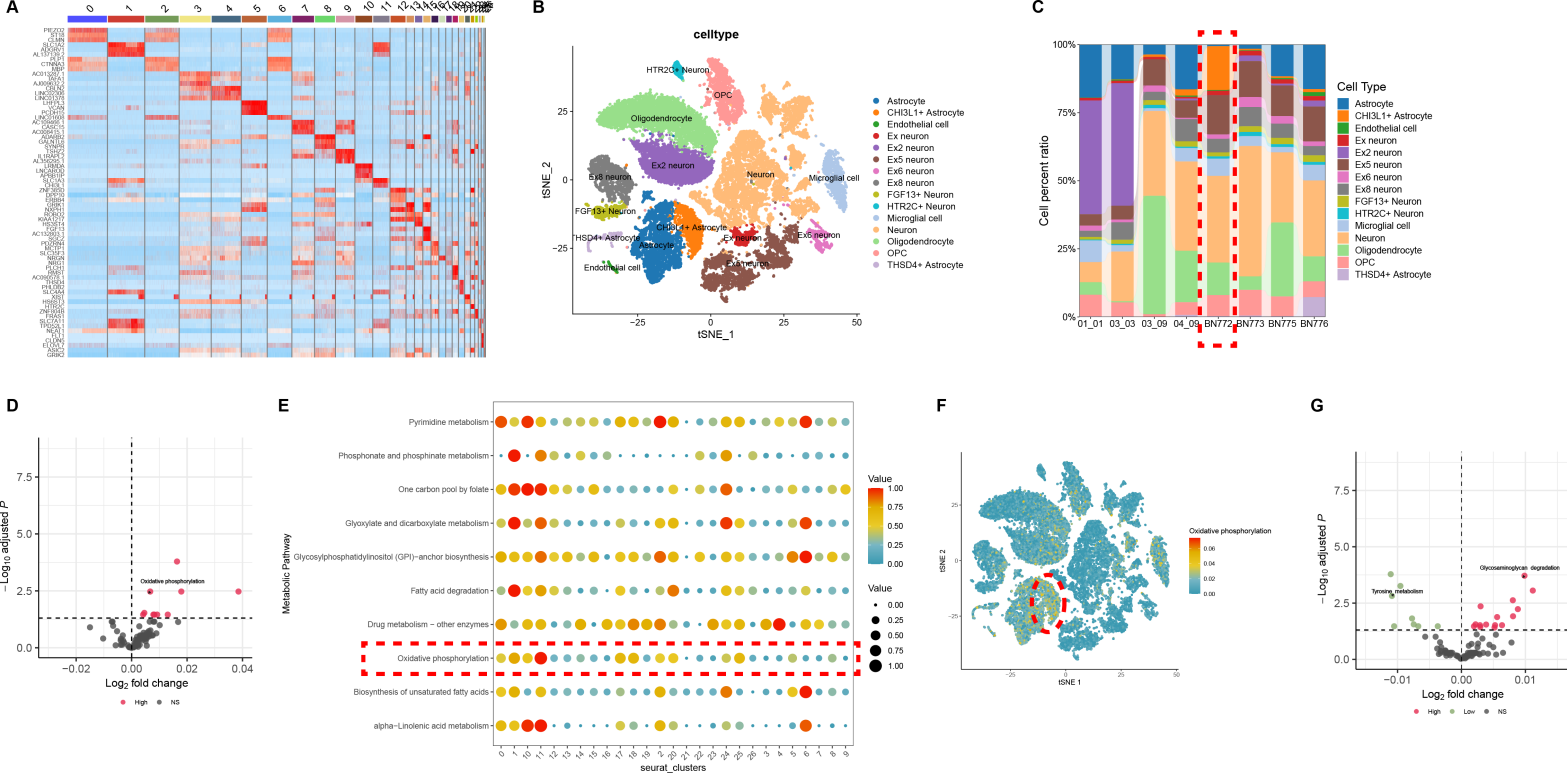
The scRNA-seq summarized the differences in COVID-19 and non-viral samples. (**A**) Top three markers of each cluster obtained from the “FindAllMarkers” function from the Seurat package (4.0.4) were shown in heatmap. (**B**) Identifying clusters of *CHI3L1* from COVID-19 samples. (**C**) The proportion of each cluster in different samples. (**D**) The scMetabolism analysis of genes from 11 clusters. (**E**) The metabolic activity analysis of brain tissues. The circle size and color darkness both represent the scaled metabolic score. (**F**) Identifying clusters of the oxidative phosphorylation pathway from COVID-19 samples. (**G**) The scMetabolism analysis of genes from the 0 cluster.

Based on the above analysis of cell clustering and functional annotation, the cell subpopulation was confirmed to consist of 15 known types, including astrocytes, CHI3L1 + astrocytes, endothelial cells, and neurons (Fig. 5A and B). After clustering using the Seurat package, the distribution of cells specifically expressing CHI3L1 was assessed, and it was found that CHI3L1 was highly expressed in the astrocytes cluster (Fig. 5B through C), which was consistent with previous research (22). Subsequently, we used scMetabolism analysis to assess metabolic activity between the 11 subpopulations and the 0 subpopulation in the COVID-19 specimens and the non-viral specimens. The results showed that astrocytes from 11 subpopulations in the COVID-19 group were in a state of metabolic activity, with a particularly prominent increase in the level of oxidative phosphorylation (Fig. 5D through F). In contrast, neurons from the 0 subpopulation exhibited decreased tyrosine metabolism and increased degradation of glycosaminoglycans (Fig. 5G). These metabolic changes are closely associated with PD, suggesting that COVID-19 infection might increase the risk of PD by affecting these metabolic pathways (15, 24, 25).

### Pseudotime analysis in COVID-19

**Fig 6:**
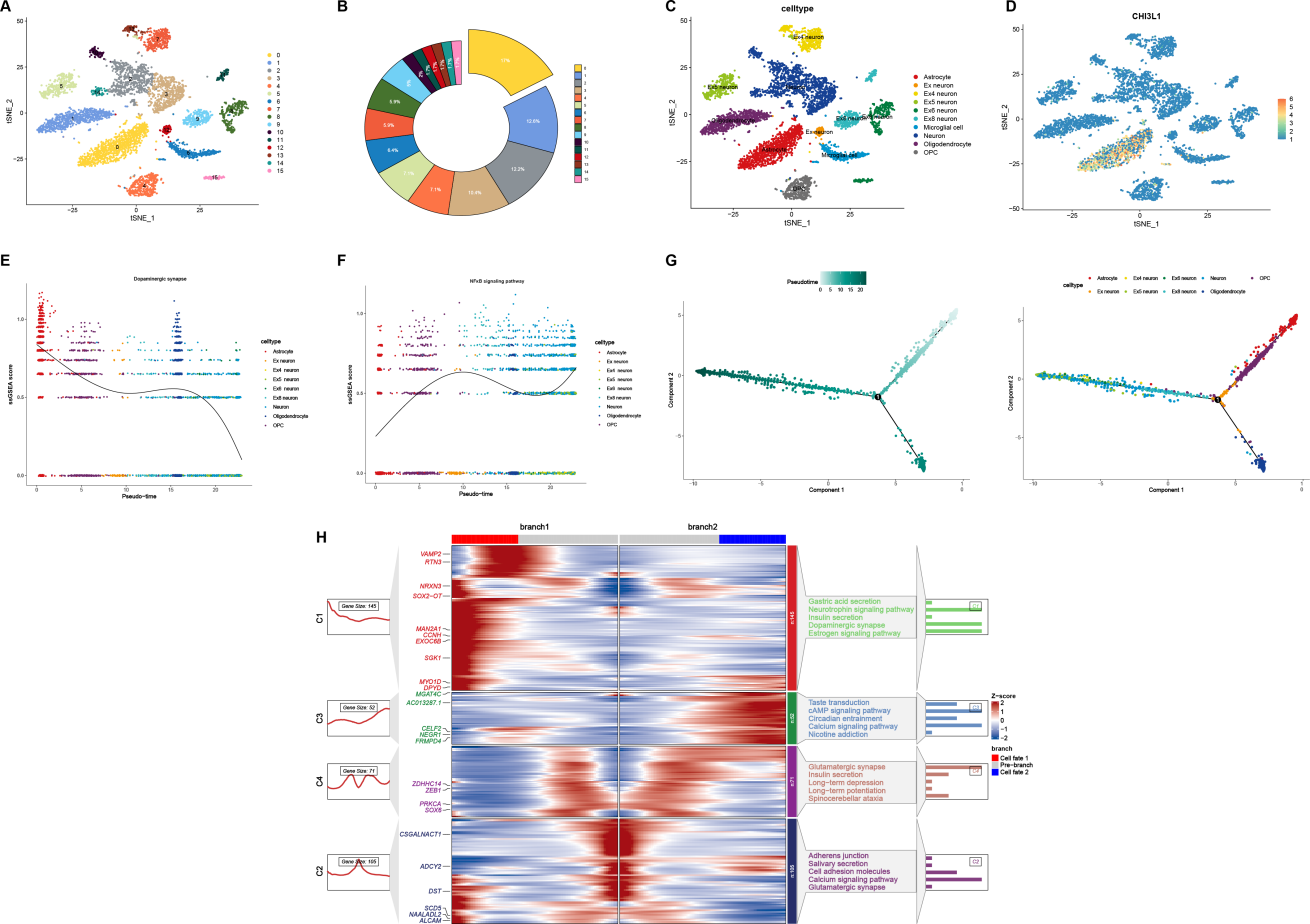
Pseudotime analysis of COVID-19 sample from 11 clusters. (**A**) Uniform manifold approximation and projection showed the clusters learned by Seurat in R. (**B**) The proportion of each cluster in COVID-19 sample. (**C**) Identifying clusters of cell types. (**D**) The mapping of *CHI3L1* in clusters. (**E and F**) Single-sample gene set enrichment analysis (ssGSEA) of dopaminergic synapse and NF-κB signaling pathway. (**G**) Trajectory analysis of astrocytes in COVID-19 sample. The mapping of cluster 11 and the pseudotime shown in DDRTree reduction in monocle2 package (2.18.0) in R (4.0.5). (**H**) The differential expression genes (DEGs) of different branches (different cell fates in panel **C**) shown in the heatmap. The top GO BP pathways of different clusters in the heatmap were listed nearby.

Upon conducting pseudotime analysis on brain tissue from COVID-19 specimens derived from subpopulation 11 (Fig. 6A through D), we observed an early activation of astrocytes with elevated CHI3L1 expression during the initial phase of infection (Fig. 6E). Concurrently, the NF-κB signaling pathway, which is linked to chronic inflammation, showed a progressive increase as COVID-19 advanced, while the activity of dopaminergic neurons diminished over time (Fig. 6E and F). Genes highly expressed in the pre-branch stage (Fig. 6G) were predominantly enriched in “Neurotrophin signaling pathway” and “Dopaminergic synapse” pathways, whereas genes related to “glutamatergic synapse” and “long-term depression” were upregulated in cell fate 2. Additionally, genes involved in the “cAMP signaling pathway” and “calcium signaling pathway” were more highly expressed in cell fate 1 (Fig. 6H). These data suggest that early CHI3L1 overexpression in astrocytes, combined with the suppression of dopaminergic neuron activity, may play a pivotal role in the elevated risk of Parkinson’s disease following COVID-19 infection.

### The inference of cell-cell communication

**Fig 7:**
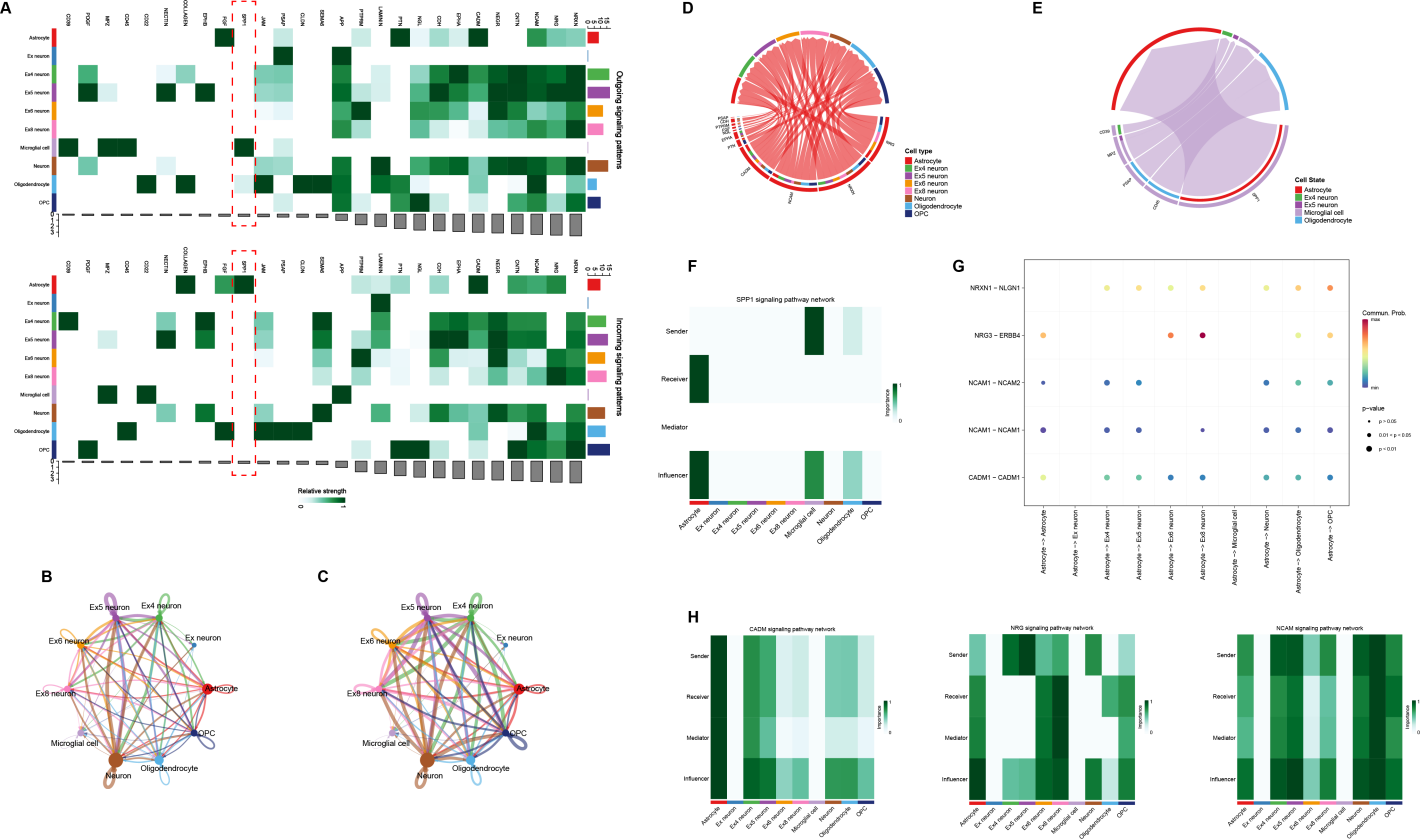
Cell-cell communication. (**A**) The outgoing and incoming signaling patterns between nerve cells. (**B**) Circle plot showing the communication numbers between interacting cells. (**C**) Circle plot showing the communication strength between interacting cells. (**D**) Chord plot showing the inferred communication network emanating from astrocytes. (**E**) Chord plot showing the inferred communication network received by astrocytes. (**F**) SPP1 signaling pathway network between microglia and astrocytes. (**G**) The dot plot showing the expression of the five genes involved in astrocytes outgoing communication. (**H**) Astrocytes and neurons communication in CADM, NRG, and NCAM signaling pathways.

We employed CellChat to systematically dissect the intercellular communication networks within distinct cell clusters identified in this scRNA-seq data set, utilizing default parameters. Our analysis revealed extensive cellular interactions mediated by 27 distinct signaling pathways (Fig. 7A). These pathways, ranked by interaction frequency and strength, highlighted astrocytes as key participants, exhibiting enhanced cell-to-cell communication (Fig. 7B and C). Notably, astrocytes were found to be highly versatile, functioning both as signal emitters and receivers, engaging in crosstalk with microglia, excitatory neurons (Ex neurons), and oligodendrocytes through a variety of signaling pathways (Fig. 7D and E). Astrocytes emerged as central hubs in many pathways, influencing both neuronal and glial populations (26). Among these, microglia-astrocyte communication via the SPP1 signaling axis was particularly prominent (Fig. 7A and F). Furthermore, astrocytes engaged with specific Ex neuron subtypes (Ex4, Ex5, Ex6, and Ex8) through molecules such as CADM1, NCAM1, and NRG (Fig. 7G and H). Crosstalk with oligodendrocytes and oligodendrocyte progenitor cells, essential for maintaining the integrity of myelinated axons, was mediated via the NRXN1 pathway (Fig. 7G and H) (27). This intricate network underscores the pivotal role of astrocyte-mediated communication in regulating neuronal excitability, synaptic plasticity, and immune responses in the brains of COVID-19 patients and provides mechanistic insights into the potential link between COVID-19 infection and the onset of PD.

## DISCUSSION

Globally, 55%–60% of individuals infected with SARS-CoV-2 progress to COVID-19, with an estimated 10%–45% continuing to experience symptoms 3 months post-infection (28). Evidence supports that SARS-CoV-2 can compromise the integrity of the human blood-brain barrier and cause neuropathological damage (14, 29). Neurological complications are widespread, with up to 85% of patients reporting acute and subacute manifestations, not limited to those with severe COVID-19 but extending to individuals with mild or asymptomatic infections (8). Research documented the emergence of diverse forms of parkinsonism following COVID-19 infection, with potential mechanisms linked to SARS-CoV-2-mediated neuroinflammation and nigrostriatal dopaminergic impairment (30). To further investigate the potential relationship between COVID-19 and PD, this study analyzed transcriptomic profiles from 40 samples across two GEO data sets, with the objective of identifying key molecular signatures implicated in both conditions. By integrating these large-scale data sets, the study aimed to uncover shared biological pathways and molecular targets that could provide insights into the common mechanisms driving COVID-19 and PD pathogenesis.

Through comprehensive bioinformatic analyses, we identified a total of 77 common DEGs shared between COVID-19 and PD. GO and KEGG enrichment analyses indicated that these common DEGs were significantly associated with inflammation-related pathways, wound healing processes, and receptor-ligand interactions. Our results corroborate previous studies that highlight neuroinflammation as a common event linking PD and COVID-19 (31, 32). Additionally, the observation that olfactory dysfunction is a shared characteristic of both PD and COVID-19 patients further implies the existence of common pathways (33). In PD, olfactory impairment primarily results from α-synuclein misfolding, leading to the degeneration of dopaminergic neurons in the substantia nigra compacta (SNc) and the formation of Lewy bodies (34). Similarly, in COVID-19 patients, the olfactory epithelium shows infiltration of inflammatory cells and production of pro-inflammatory cytokines and chemokines, which activate microglia and astrocytes, ultimately contributing to olfactory dysfunction (33). SARS-CoV-2 not only invades the SNc and striatum but also targets angiotensin-converting enzyme 2 (ACE2) receptors in the midbrain nigrostriatal system, ultimately leading to damage to the dopaminergic system and exacerbating PD-related motor symptoms (33, 35). Consistent with these reports, our study employing GSEA also revealed activation of pro-inflammatory mediators and suppression of dopaminergic neuron function in brain samples from both COVID-19 and PD. These findings reinforce the notion that neuroinflammatory responses—characterized by the production of inflammatory cytokines and chemokines, extensive glial activation, and dysregulation of neuroimmune metabolism—may contribute to the progression of both COVID-19 and PD.

We analyzed shared DEGs using Cytoscape and identified CHI3L1 as the sole significantly altered gene common to both COVID-19 and PD, suggesting its potential role as a shared target in the pathogenesis of these conditions. Previous studies have established that CHI3L1 is a secreted glycoprotein that regulates various essential biological processes in the periphery, including oxidative damage, apoptosis, inflammasome activation, M2 macrophage differentiation, extracellular matrix regulation, and tissue scarring (22, 36). It plays a critical role in responses to pathogens, antigens, and oxidative stress, as well as in inflammation, tissue repair, and remodeling (22). In the central nervous system, CHI3L1 is primarily synthesized and secreted by reactive astrocytes, where it participates in modulating inflammatory responses and activating gene transcription signaling pathways (22). Increased expression of CHI3L1 has been observed in various neurodegenerative diseases, and studies have confirmed that CHI3L1 is involved in synaptic plasticity and cognitive dysfunction in mouse models of Alzheimer’s disease (36, 37). Similarly, alterations in CHI3L1 levels have been detected in the cerebrospinal fluid of PD patients, with elevated levels correlating with neuroglial responses, axonal damage, and poorer cognitive performance (36). Moreover, changes in CHI3L1 expression levels are also considered to be related to the severity of COVID-19 (20). Consistent with previous findings, our scRNA-seq results from COVID-19 brain tissue samples indicated a dramatic upregulation of CHI3L1, exhibiting significant heterogeneity, with a high proportion of CHI3L1-positive astrocytes. Pseudotime analysis further confirmed that astrocytes are among the first cells activated early in COVID-19 infection, showing a marked increase in oxidative phosphorylation levels. Moreover, we also observed dysregulation of excitatory neurotransmission and synaptic plasticity following COVID-19 infection, suggesting that CHI3L1 may be a key molecule involved in the impairment of neural communication and cognitive deficits in both COVID-19 and PD. Interestingly, related studies in elderly COVID-19 patients have shown elevated CHI3L1 expression, which serves as an activating factor for ACE2 and spike protein-processing proteases (38). This finding also provides a theoretical basis for the involvement of CHI3L1 in the pathogenic mechanisms of both PD and COVID-19, and further studies are needed.

Cell-to-cell communication is recognized as a fundamental mechanism for the transmission of information between cells, enabling appropriate physiological responses (39). Through regulated cell-to-cell interactions, various cell types contribute to the maintenance of homeostasis and overall health, providing defense against external threats such as viral infections (40). It is the dynamic interplay between neurons and glial cells that endows synaptic strength with adaptability and plasticity (41). In this study, we analyzed scRNA-seq data sets derived from COVID-19 patient samples to elucidate the cell communication networks among distinct cellular clusters. Our findings reveal that astrocytes exhibit active intercellular communication profiles, acting as both signal transmitters and receivers in complex interactions with microglia, excitatory neurons, and oligodendrocytes. This suggests that COVID-19 may exert profound effects on neural circuitry plasticity through these intercellular interactions (42). Further analysis indicates that secreted phosphoprotein 1 (SPP1), a signaling molecule between microglia and astrocytes, displays enhanced communication functions in the brains of COVID-19 patients. This enhancement implies that SPP1 may modulate microglial phagocytic activity and astrocytic-mediated synaptic regeneration, with an imbalance in these processes potentially underpinning the impairment of neuronal circuits and functions associated with COVID-19 (43, 44). In addition, astrocytes can interact specifically with distinct subtypes of excitatory neurons through CADM1, NCAM1, and NRG, suggesting that they may play a critical role in myelination and inflammatory regulation during the progression of COVID-19 (45, 46). Moreover, the communication between astrocytes and oligodendrocytes, as well as oligodendrocyte precursor cells, appears to depend on NRXN1-NLGN1 ligand-receptor interaction patterns, suggesting that SARS-CoV-2 may be involved in regulating the differentiation of astrocytes (47). It is evident that this complex astrocyte-mediated communication network highlights their important roles in regulating neuronal excitability, synaptic plasticity, and immune responses, providing valuable mechanistic insights into the potential link between COVID-19 infection and the development of PD.

This study has several limitations. We employed a retrospective design based on publicly available data, and the findings have yet to be validated through laboratory experiments using human brain tissue specimens or animal models. Furthermore, the databases lacked information on different stages or subtypes of PD, and demographic characteristics may have been inconsistent. Despite these limitations, the comparison of these two databases enabled the identification of an association between COVID-19 infection and the subsequent development of PD.

### Conclusion

In summary, our findings demonstrate the critical role of neuroinflammation and dopaminergic neuron damage in the shared pathogenesis of COVID-19 and PD. CHI3L1 emerged as a key target, highlighting its potential involvement in modulating neuroinflammatory pathways and synaptic plasticity. The functional significance of CHI3L1, as well as its pathological relevance, warrants further investigation through larger studies. Additionally, the active intercellular communication between astrocytes, microglia, and excitatory neurons underscores the profound impact of COVID-19 on neural circuitry. These results provide important insights into the mechanisms driving the neurodegenerative consequences of COVID-19, reinforcing the need for continued exploration of therapeutic interventions and the long-term neurological effects of viral infection.

## Data Availability

The bulk-seq expression profile and relevant clinical data of PD and COVID-19 samples at various stages were collected from the Gene Expression Omnibus (GEO; https://www.ncbi.nlm.nih.gov/geo/) with accession numbers GSE184950 and GSE182299.
